# Electronic cigarettes: Emerging trends and research hotspots

**DOI:** 10.18332/tid/118719

**Published:** 2020-03-16

**Authors:** Qiang Zhang, Xinyue Fan, Yuanyi Yue, Rui Zheng

**Affiliations:** 1 Department of Pulmonary and Critical Care Medicine, Shengjing Hospital of China Medical University, Shenyang, China; 2 Student Affairs Department, Shengjing Hospital of China Medical University, Shenyang, China; 3 Department of Gastroenterology Medicine, Shengjing Hospital of China Medical University, Shenyang, China

**Keywords:** electronic cigarette, bibliometric analysis, co-word analysis, strategic diagram analysis, social network analysis

## Abstract

**INTRODUCTION:**

Research on electronic cigarettes is an emerging field, with the number of articles in this field noted to have grown exponentially over recent years. We used a bibliometric analysis method (co-word analysis) to analyze the emerging trends and research hotspots in this field.

**METHODS:**

Publication data on electronic cigarettes from 2010 to 2018 were retrieved and downloaded from the PubMed database. Theme trends and knowledge structures were analyzed on the relevant research fields of electronic cigarettes by using a biclustering analysis, strategic diagram analysis, and social network analysis methods. Research hotspots were extracted and compared from three periods.

**RESULTS:**

Core topics that have continuously develop between the years 2010 and 2018 include: tobacco use cessation devices; tobacco products; tobacco use cessation devices/adverse effects; smoking prevention and adverse effects; electronic nicotine delivery systems/economics; and public health. Some currently undeveloped topics that could be considered as new future research directions include: tobacco use disorder/therapy; tobacco use disorder/epidemiology; students/psychology; students/statistics and numerical data; adolescent behavior/psychology; nicotine/toxicity; nicotinic agonists/administration and dosage; and electronic nicotine delivery systems/legislation and jurisprudence.

**CONCLUSIONS:**

Results suggest that some currently immature topics in strategic coordinates and emerging hotspots in social network graphs can be used as future research directions.

## INTRODUCTION

An electronic cigarette (e-cigarette) refers to a cigarette consisting of a battery, an evaporation heating device, and a tobacco tube containing a liquid smoking product. The nicotine-containing tobacco liquid can be turned into vapor by nebulization for the user to inhale^1^. There is growing evidence that, even though e-cigarettes may produce fewer toxic substances than traditional cigarettes, they may still pose health risks to smokers and people around them^2,3^. The long-term effects of e-cigarettes on health are not currently apparent. Moreover, there is insufficient evidence to show that such products may assist people to quit traditional cigarettes^4^.

A large number of recent studies have shown that, from a toxicological standpoint, e-cigarettes as a substitute for nicotine, compared to traditional smoking, may aid in improving public health^5,6^. However, e-cigarettes may contribute to adverse reactions in the respiratory system^7,8^, cardiovascular system^9^, liver^10^, and nervous system^11^. Ever since e-cigarettes became popular among adolescents, the number of studies on e-cigarettes has increased^12,13^. In recent years, researchers have become increasingly concerned about tobacco-use disorders^14^. Some studies have shown that comprehensive interventions are needed to help protect adolescents’ mental health^15,16^. With the development of bibliometrics comes its widespread use in health topics. However, there is only a limited amount of bibliometric analyses that focus on tobacco, and we have found only two that focused on e-cigarettes^17,18^. This study used a co-word analysis instead of the co-citation analysis applied by the above two articles, to examine trends in e-cigarette research.

## METHODS

### Data collection and bibliographic matrix setup

This study used the Bibliographic Item Co-Occurrence Matrix Builder (BICOMB) to extract data for the three periods 2010–2012, 2013–2015 and 2016–2018, from PubMed including journals, countries, authors, and major MeSH (*Medical Subject Headings*) terms/subheadings from which were calculated the number of high-frequency major MeSH terms/subheadings, using the Donohue equation^19^:

$$T=(1+\sqrt{\left(1+8\text{i}\right)}/2$$

where i is the number of major MeSH terms/subheadings that have occurred only once. BICOMB was then used to generate the term-source article and co-occurrence matrix as the data basis for our subsequent bibliometric analysis. Ethical approval was not necessary according to the Ethics Committee, Medical University of Lodz, as this was not an experimental study.

### Biclustering analysis

The current study used a biclustering analysis of the high-frequency major MeSH terms/subheadings and PubMed unique identifiers on e-cigarette related publications retrieved from PubMed^20^. Biclustering analysis was conducted to classify the major MeSH terms/subheadings based on the term-source article matrix. In this work, we use word groupings of high-frequency major MeSH terms/subheadings as keywords. The gCLUTO software was used to create a clustering mountain visualization and to construct a visual matrix using the repeated bisection method. In a clustering mountain visualization, each peak represents a cluster and each peak position, volume, height and color correspond to the relevant cluster data, enabling an easy, intuitive observation of the structure of each research hotspot in the field of e-cigarettes. The color of the mountain peaks is proportional to the standard deviation within the class. Red represents low standard deviation and blue represents a high standard deviation, but only the color of the peak is significant. The distance between the peaks expresses the similarity between clusters. This result proposes and analyzes research priorities in related fields.

### Strategic diagram analysis

GraphPad 5 software was used to create a strategic diagram to analyze the mutual influence within and between domains of e-cigarettes. The strategy diagram has two axes: the y-axis represents density, indicating the ability to maintain and develop itself; and the x-axis represents the centrality, indicating the degree of interaction between the indicated field and others^21^. Based on the high-frequency significant MeSH terms co-occurrence matrix and biclustering analysis, we can calculate the centrality and density of each cluster to describe the internal connection and interaction between clusters in a research field. Density is the closeness of the keywords in each cluster, which indicates the ability of the cluster to maintain and develop itself, and centrality is the closeness between the keywords of each cluster and of other clusters, which indicates the interaction effect. The two axes produce four quadrants with major MeSH terms/subheadings assigned to different quadrants based on the results of the biclustering analysis. By comparing the strategic diagrams of the three study time periods, the evolution of a field of e-cigarettes can be estimated.

### Social network analysis

The current study used Ucinet 6.0 (Analytic Technologies Co., KY, USA) to construct a social network analysis (SNA) in order to further analyze the knowledge structure of the e-cigarette field. Applying NetDraw 2.084, the major MeSH terms/subheadings network was able to be presented in a 2D map. The nodes represent the major MeSH terms/subheadings, and the links represent their co-occurrence frequencies. Each significant MeSH term/subheading can be evaluated by three parameters, namely, degree, closeness, and betweenness. Degree refers to the number of other nodes directly connected to a node. The higher the degree centrality of a node, the more critical it is to indicate its position in the network. Betweenness indicates the number of shortest paths through a node. The more times a node acts as an intermediary node, the higher is its significance in evaluating the importance of a node in the network. Closeness reflects the closeness between a node and other nodes in the network. It is the sum of the reciprocal of the shortest distances from a specific node to all other nodes in the network. This means that the higher the closeness, the shorter is the distance from this node to other nodes in the network. It is worth noting that the betweenness index was chosen as an evaluation index for the in-depth study of the e-cigarette field.

## RESULTS

### Characteristics of e-cigarette related publications

We retrieved and analyzed 54, 968, and 2406 publications for each period, respectively. The number of articles related to e-cigarettes has increased from 8 in 2010 to 988 in 2018, nearly 120 times during the past nine years. Table 1 lists the top ten countries by number of publications, while Table 2 lists the top ten journals (by number of articles), for the three periods. We suggest that these represent the core changes in the field of e-cigarettes over the past nine years. England was the country with the most published articles in the three periods, followed by the United States. China ranked tenth in the first period, but had no special contribution in the other two time periods.

In the first time period (2010–2012), the top three journals that published articles on e-cigarettes were: *Tobacco Control; Nicotine & Tobacco Research;* and *Przeglad Lekarski;* which accounted for 27.3% of the total publications. In the second time period (2013–2015), *Nicotine & Tobacco Research* ranked first, and *Przeglad Lekarski* was replaced by *BMJ (Clinical Research ed.).* In the third time period (2016-2018), the top three journals were: *Tobacco Control; Nicotine & Tobacco Research;* and *Addictive Behaviors.* Overall, *Nicotine & Tobacco Research* has published the most articles in the field of e-cigarettes over the last nine years.

**Table 1:** Top ten countries by number of publications in the field of e-cigarettes for the three time periods 2010–2012, 2013–2015 and 2016–2018

*Rank*	*2010–2012 n (%)*	*2013–2015 n (%)*	*2016–2018 n (%)*
1	England 28 (45.1)	England 458 (41.9)	England 906 (37.6)
2	United States 17 (27.4)	United States 313 (28.6)	United States 795 (33.0)
3	Poland 3 (4.8)	Switzerland 81 (7.4)	Netherlands 201 (8.3)
4	New Zealand 3 (4.8)	Netherlands 64 (5.8)	Switzerland 155 (6.4)
5	Netherlands 3 (4.8)	Canada 22 (2.0)	Ireland 96 (3.9)
6	Italy 2 (3.2)	Germany 21 (1.9)	Germany 43 (1.7)
7	Germany 2 (3.2)	Ireland 17 (1.5)	Canada 33 (1.3)
8	Korea (South) 1 (1.6)	Australia 17 (1.5)	Australia 23 (0.9)
9	Czech Republic 1 (1.6)	France 17 (1.5)	Greece 21 (0.8)
10	China 1 (1.6)	Norway 16 (1.4)	France 17 (0.7)
Total	61 (98.3)	1026 (94.0)	2290 (95.2)

**Table 2:** Top ten journals by number of publications in the field of e-cigarettes for the three time periods 2010–2012, 2013–2015 and 2016–2018

*Rank*	*2010–2012 n (%)*	*2013–2015 n (%)*	*2016–2018 n (%)*
1	Tobacco Control 9 (14.5)	Nicotine & Tobacco Research 89 (8.1)	Nicotine & Tobacco Research 160 (6.6)
2	Nicotine & Tobacco Research 5 (8.0)	Tobacco Control 74 (6.7)	Tobacco Control 135 (5.6)
3	Przeglad Lekarski 3 (4.8)	BMJ (Clinical Research ed.) 46 (4.2)	Addictive Behaviors 121 (5.0)
4	Addiction (Abingdon, England) 3 (4.8)	Addiction (Abingdon, England) 44 (4.0)	International Journal of Environmental Research and Public Health 84 (3.4)
5	Chest 2 (3.2)	International Journal of Environmental Research and Public Health 32 (2.9)	Drug and Alcohol Dependence 78 (3.2)
6	The New Zealand Medical 2 (3.2)	Journal Addictive Behaviors 26 (2.3)	Addiction (Abingdon, England) 48 (1.9)
7	The Journal of Adolescent Health 2 (3.2)	PloS One 23 (2.1)	Preventive Medicine 46 (1.9)
8	MMW Fortschritte der Medizin 2 (3.2)	American Journal of Preventive Medicine 21 (1.9)	Regulatory Toxicology and Pharmacology: RTP 46 (1.9)
9	Inhalation Toxicology 2 (3.2)	Revue Medicale Suisse 20 (1.8)	PloS One 43 (1.7)
10	American Journal of Preventive Medicine 2 (3.2)	Tidsskrift for den Norske Laegeforening: Tidsskrift for Praktisk Medicin, ny Raekke 16 (1.4)	American Journal of Preventive Medicine 41 (1.7)
Total	32 (51.6)	391 (35.8)	802 (33.3)

### Research hotspots in MeSH term clusters

According to the publications retrieved for the three time periods, there were respectively 15, 26 and 49 high-frequency major MeSH terms/subheadings (Supplementary file, Table S1) with a total frequency of occurrence of 49.6%, 49.5% and 50.4%, respectively. We considered these as the research hotspots for the three time periods. A biclustering analysis leads to a division of MeSH terms into 4, 3 and 5 clusters for each period, respectively (Table 3). Biclustering analysis results of the high-frequency major MeSH terms/subheadings in the field of e-cigarettes for the three time periods are also listed. Mountain and matrix visualizations of major MeSH terms/subheadings were also conducted (Supplementary file, Figures S1, S2 and S3). The mountain visualization showed cluster 0 in 2010–2012, cluster 0 in 2013–2015, and clusters 2 and 4 in 2016–2018 as the most significant, respectively.

**Table 3:** Cluster analysis of high-frequency MeSH terms/subheadings of e-cigarettes for the three time periods 2010–2012, 2013–2015 and 2016–2018

*Period*	*Cluster*	*Ranknumber of MeSH terms/subheadings*	*Cluster analysis*
**2010–2012**	0	1, 2, 7, 8, 12	Nicotine/administration & dosage Smoking Cessation/methods
	1	3, 11, 15	Smoking Prevention and Adverse Effects Tobacco Use Cessation Devices/adverse effects
	2	6, 10, 15	Tobacco Products Tobacco Use Cessation Devices
	3	4, 5, 9, 14	3. Smoking Cessation/legislation & jurisprudence
**2013–2015**	0	2, 5, 7, 9, 12, 15, 16, 17, 27	Electronic Nicotine Delivery Systems/statistics & numerical data Tobacco Use Cessation Devices Smoking Cessation/smoking/psychology
	1	1, 10, 13, 14, 18, 20, 23	Electronic Nicotine Delivery Systems Tobacco Products
	2	3, 4, 6, 8, 11, 19, 21, 22, 24, 25, 26	Tobacco Use Cessation Devices/adverse effects Electronic Nicotine Delivery Systems/trends and economics Smoking Prevention and Adverse Effects Public Health
**2016–2018**	0	5, 10, 15, 19, 26, 28, 30, 38	1. Electronic Nicotine Delivery Systems/adverse effects
	1	7, 8, 14, 20, 25, 29, 34, 44, 48	Students/psychology Electronic Nicotine Delivery Systems/psychology
	2	4, 9, 11, 13, 27, 37, 40, 43, 45, 49	Electronic Nicotine Delivery Systems/economics Electronic Nicotine Delivery Systems/legislation & jurisprudence Tobacco Use Disorder/therapy
	3	1, 12, 16, 17, 22, 24, 35, 39, 42	Electronic Nicotine Delivery Systems/instrumentation Tobacco Products Public Health
	4	2, 3, 6, 18, 21, 23, 31, 32, 33, 36, 41, 46, 47	Students/statistics & numerical data Cigarette Smoking/epidemiology Electronic Nicotine Delivery Systems/statistics & numerical data

MeSH: Medical Subject Headings.

### Theme trends of e-cigarettes

**Figure 1:**
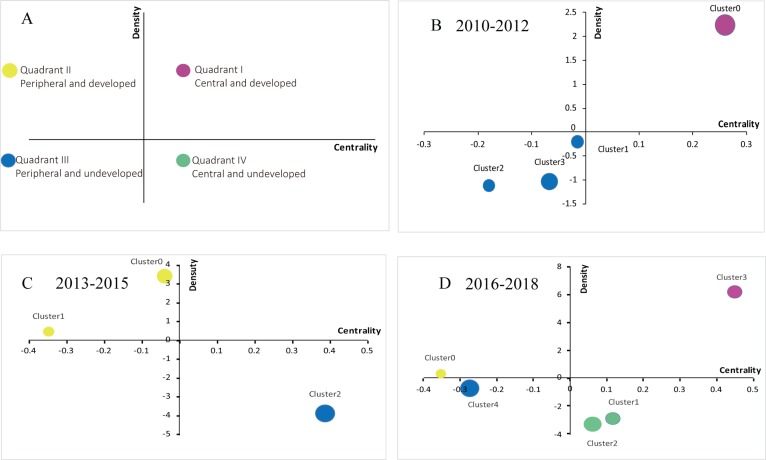
Strategic quadrant diagrams for each period of the e-cigarette field A) The meaning of strategic diagram; B) Strategic diagram for e-cigarettes in 2010–2012; C) Strategic diagram for e-cigarettes in 2013–2015; D) Strategic diagram for e-cigarettes in 2016–2018. Clusters in each strategic diagram refer to the biclustering results presented in Table 3. The size of a signal node represents the number of major MeSH terms/subheadings involved in each cluster.

Callon^22^ explains the meaning of strategic diagrams (as shown in Figure 1). Motor-themes are located in quadrant I (top right), with high density and centrality. The topics within this quadrant are generally central themes in the e-cigarette field and are closely related to other topics. Specialized themes are located in quadrant II (upper left corner) and topics within this quadrant are at the periphery of the field but are emerging. The topics in quadrant III (bottom left corner) are at the periphery of the field and undeveloped, while the topics in quadrant IV (bottom right corner) are at the center of the field but undeveloped. The size of each circle is proportionate to the number of high-frequency major MeSH terms/subheadings within it.

In the first time period, only cluster 0 was in quadrant I, suggesting that Nicotine/administration & dosage, and Smoking Cessation/methods, were at the core of the e-cigarette field during this period, with high concentration and good development. Clusters 1, 2 and 3 were in the third quadrant, suggesting that research on Smoking Prevention and Adverse Effects, Tobacco Use Cessation Devices/adverse effects, Tobacco Products, Tobacco Use Cessation Devices, and Smoking Cessation/legislation & jurisprudence were not yet mature and were at the edge of the e-cigarette field.

In the second time period, new topics emerged, namely: Electronic Nicotine Delivery Systems/statistics & numerical data; Smoking Cessation/smoking/psychology; Electronic Nicotine Delivery Systems; Electronic Nicotine Delivery Systems/trends and economics; and Public Health. Some immature topics from the previous time period evolved into mature themes, namely: Tobacco Use Cessation Devices, and Tobacco Products. Additionally, some of the marginal themes from the previous time period developed into the center of the field in the most recent time period, but are still immature, namely: Tobacco Use Cessation Devices/adverse effects; and Smoking Prevention and Adverse Effects.

Some topics remain at the center of the field in 2014–2018 from 2009–2013, e.g. Electronic Nicotine Delivery Systems/economics. New topics are, however, currently emerging, e.g. Electronic Nicotine Delivery Systems/instrumentation. Some immature themes from the previous time period developed into mature themes for the most recent time period, namely: Tobacco Products, and Public Health. It is worth noting that there are also new topics in quadrant III, which are at the edge of the e-cigarette field, such as: Students/statistics & numerical data; Cigarette Smoking/epidemiology; and Electronic Nicotine Delivery Systems/statistics & numerical data. These topics may, however, change in the future.

### Knowledge structure of e-cigarettes

**Figure 2:**
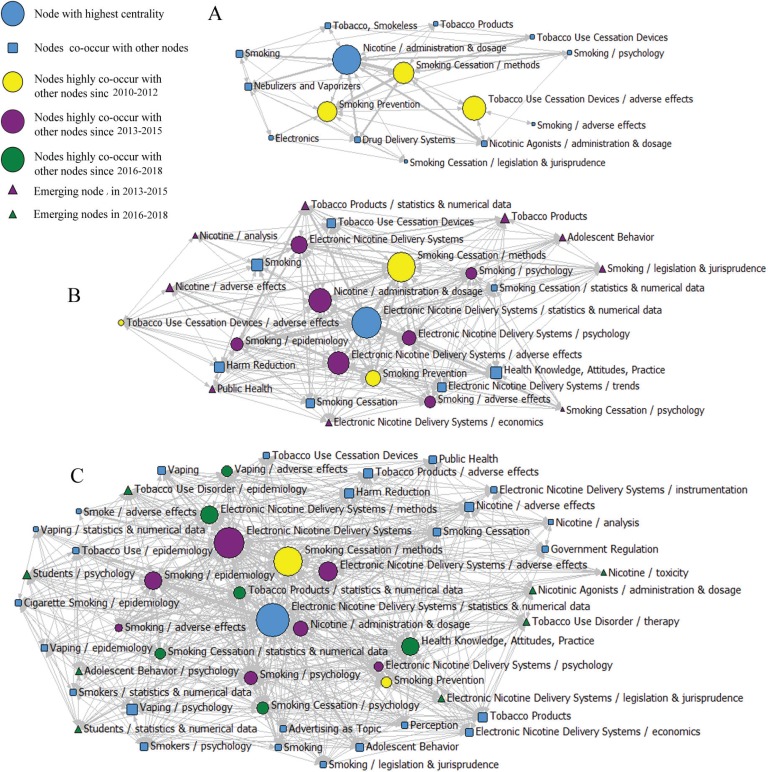
Social network analysis for MeSH terms/subheadings in e-cigarette related publications A) Social network analysis for 15 major MeSH terms/subheadings in 2010–2012; B) Social network analysis for 26 major MeSH terms/subheadings in 2013–2015; C) Social network analysis for 49 major MeSH terms/subheadings in 2016–2018. The size of the nodes indicates MeSH terms centrality. The thickness of the lines indicates the co-occurrence frequency of MeSH terms pairs.

As shown in Figure 2, the three SNA diagrams are constructed by the three indicators: degree, betweenness, and closeness; where betweenness is the index. The size of the nodes is proportional to the betweenness of these major MeSH terms/subheadings, while the thickness of the lines represents the co-occurrence frequency of MeSH terms pairs.

As demonstrated in the 2010–2012 SNA diagram, the top 15 major MeSH terms/subheadings are highly central, with Nicotine/administration & dosage having the highest betweenness (16.267), playing the most important role in the network. In addition, the closeness value is 13, indicating that it is the most closely connected to other nodes. In addition, Tobacco Use Cessation Devices/adverse effects, Smoking Cessation/methods, and Smoking Prevention, also have high degrees of betweenness, indicating that they also have key intermediary roles in the network. The average betweenness value of these three topics is 4.333.

Compared to the first three years, Electronic Nicotine Delivery Systems/statistics & numerical data has the highest level of betweenness in the second period SNA diagram and seven new major MeSH terms/subheadings emerged, including: Smoking/Psychology; Electronic Nicotine Delivery Systems/psychology; Smoking/adverse effects; Electronic Nicotine Delivery Systems/adverse effects; Smoking/epidemiology; Electronic Nicotine Delivery Systems; and Nicotine/administration & dosage. At the same time, nine new nodes emerged at the edge of the network, including: Nicotine/analysis; Nicotine/adverse effects; Public Health; Electronic Nicotine Delivery Systems/economics; Smoking Cessation/psychology; Smoking/legislation & jurisprudence; Adolescent Behavior; Tobacco Products; and Tobacco Products/statistics & numerical data. These are considered as emerging hotspots in the field of e-cigarettes in 2013–2015.

As demonstrated in the 2014–2018 SNA diagram, there are six new major MeSH terms/subheadings, including: Vaping/adverse effects; Electronic Nicotine Delivery Systems/methods; Tobacco Products/statistics & numerical data; Smoking Cessation/statistics & numerical data; Smoking Cessation/psychology; and Health Knowledge Attitudes and Practice. There are eight new nodes at the edge of the network, namely: Tobacco Use Disorder/therapy; Tobacco Use Disorder/epidemiology; Students/psychology; Students/statistics & numerical data; Adolescent Behavior/psychology; Nicotine/toxicity; Nicotinic Agonists/administration & dosage; and Electronic Nicotine Delivery Systems/legislation & jurisprudence. These were emerging hotspots in the field of e-cigarettes in the period 2014–2018.

## DISCUSSION

As our knowledge of e-cigarettes continues to increase, the amount of related research also continues to grow and e-cigarette research has become an emerging field. Using a biclustering analysis, strategic diagrams, and social network analysis diagrams, we analyzed, in detail, the evolution of thematic trends and knowledge structures in the field of e-cigarettes over the past nine years. This is the first time co-word analysis was used to analyze trends in this field.

The current study examines e-cigarette publications, globally, by comparing three time periods in the past nine years (2010-2018). Over that period of time, the number of publications related to e-cigarettes has grown rapidly, with England and United States leading the way with the most published articles. *Nicotine & Tobacco Research,* and *Tobacco Control,* are the journals that have published the most articles, however the current study only analyzed the number of e-cigarette articles published in a journal, which is greatly affected by the total number of articles published in a journal. We note that although some journals have a smaller total volume of articles, they also publish in the field of e-cigarettes and have considerable influence, such as *Tobacco Induced Diseases,* and *Tobacco Prevention and Cessation* etc.

Strategic diagrams were used to analyze the theme trends of publications of three time periods. In the first time period (2010–2012), only cluster 0 is in quadrant I, which includes Nicotine/administration & dosage, and Smoking Cessation/methods. We consider these two themes to be research hotspots in the field of e-cigarettes during this period. The research in this period suggests that there is a need for more effective drugs to help smokers quit smoking. During this period, some important research focused on smoking cessation methods and compared various methods including nicotine replacement therapy (NRT), bupropion and varenicline, and nortriptyline and clonidine^23^. Clusters 1, 2 and 3 are in quadrant III. Research in these clusters focuses on: Smoking Prevention and adverse effects; Tobacco Use Cessation Devices/adverse effects; Tobacco Products; Tobacco Use Cessation Devices; and Smoking Cessation/legislation & jurisprudence. These topics are immature and are located in the periphery of e-cigarette research during this period. They may gradually shift to a central and/or mature position, if further research is undertaken. Studies have focused on adverse effects of smoking, and some have now been widely recognized, e.g. smoking cigarettes is the strongest risk factor for chronic obstructive pulmonary disease (COPD)^24^ and remains the primary risk factor also for lung cancer^25^. Legislators are increasingly recognizing the dangers of tobacco products as well as the importance of tobacco control and protecting the public from the harm of tobacco^26^. With the development of e-cigarettes, there is increased concern about their safety as cessation devices, for which there is significant debate; however others have noted that they may provide a potential to quit smoking^27^.

In the second time period (2013–2015), Tobacco Use Cessation Devices, and Tobacco Products, were noted as two themes gradually maturing. In the last period (2016–2018), researchers had high hopes for the role of Tobacco Use Cessation Devices in smoking cessation. In this period, research was more extensive, for example, with the aim to apply large cross-sectional surveys to assess the effectiveness of e-cigarettes as an aid to smoking cessation^28^, or to quantify how smokers evaluate the attributes of e-cigarettes^29^. In addition to paying attention to the harm of Tobacco Products, it was also found that e-cigarettes also have the potential to do harm because they contain nicotine, which is addictive and can cause adverse reactions^30^. Therefore, both tobacco products and e-cigarettes should be treated with caution^31^. The topics of Tobacco Use Cessation Devices/adverse effects, and Smoking Prevention and adverse effects, gradually developed into the center of the field of e-cigarettes while the adverse reactions of e-cigarettes attracted more attention. Additionally, there were new and immature topics during this period, such as Electronic Nicotine Delivery Systems/economics, and Public Health. Due to increasing attention, these two new keywords have been continuously developed.

The strategic diagram for the third time period (2016–2018) describes the knowledge structure of the e-cigarette field and provides a large amount of information on emerging, prominent research. Custer 0, Electronic Nicotine Delivery Systems/adverse effects have been developed at this stage. With the popularity of e-cigarettes growing, there was an increase in experiments *in vivo* and *in vitro* that have demonstrated the harm of e-cigarettes^32–34^. The three previously immature topics of Electronic Nicotine Delivery Systems/economics, Public Health, and Tobacco Products, evolved into mature topics. The journal Tobacco Control published several articles about Electronic Nicotine Delivery Systems/economics, and Public Health, in succession^35–37^. The journal Tobacco Prevention and Cessation had a special supplement on vape shops^38,39^. Clusters 1, 2 and 4, including: Students/psychology; Electronic Nicotine Delivery Systems/psychology; Electronic Nicotine Delivery Systems/legislation & jurisprudence; Tobacco Use Disorder/therapy; Students/statistics & numerical data; Cigarette Smoking/epidemiology; and Electronic Nicotine Delivery Systems/statistics & numerical data, are immature and need further research. During this period, there is an increasing number of studies on the epidemiology of tobacco and statistical analysis of e-cigarettes^40^. People are paying more attention to the connection between smoking and psychology, particularly the impact of e-cigarettes on youth psychology. In addition, from a social perspective, people also attach great importance to the legislation and jurisprudence governing e-cigarettes^41^.

Three SNA diagrams were made according to the high-frequency MeSH terms/subheadings. In these three time periods, 4, 10 and 16 major MeSH terms/subheadings, respectively, had a high degree of centrality. Nicotine/administration & dosage in the first time period, and Electronic Nicotine Delivery Systems/statistics & numerical data in the second and third periods are at the center of the SNA diagrams and have the greatest number of direct connections to other nodes, suggesting the most significant impact during each period.

In addition, in the second period, there are nine MeSH terms at the edge of the network that are new and immature. Among these, Nicotine/analysis, Nicotine/adverse effects, and Public Health became developed in the third period. We can consider these MeSH terms as emerging hotspots in the second period (2013–2015). Similarly, Tobacco Use Disorder/therapy, Tobacco Use Disorder/epidemiology, Students/psychology, Students/statistics & numerical data, Adolescent Behavior/psychology, Nicotine/toxicity, Nicotinic Agonists/administration & dosage, and Electronic Nicotine Delivery Systems/legislation & jurisprudence can be considered as hotspots in the most recent of the analyzed years (2016-2018).

### Strengths and limitations

To the best of our knowledge, the current study is the first to use a co-word analysis method to perform a comprehensive analysis of e-cigarette publications. The e-cigarette field is constantly evolving, and there will be more in-depth research in the future. It is our contention that the emerging hot issues mentioned above can guide clinicians and researchers to develop new projects in the e-cigarette area. At the same time, this research has certain limitations. The first is that we only searched for journals, excluding comments and other types of literature, and perhaps missed some research hotspots. Secondly, co-word analyzes high-frequency MeSH terms only, which may affect the results of the cluster analysis as our results are based on the number of articles published in each medium and not the impact of each article. Moreover, in the future, we could use a variety of databases for analysis, such as Cochrane, Embase, clinical trials.gov, some guidelines could also be searched, as well as manual searching for grey literature.

## CONCLUSIONS

We applied a biclustering analysis, strategic diagram and SNA methods, to analyze high-frequency MeSH terms, and conducted a co-word analysis on the field of e-cigarettes. This research shows that Tobacco Use Cessation Devices, Tobacco Products, Tobacco Use Cessation Devices/adverse effects, Smoking Prevention and adverse effects, Electronic Nicotine Delivery Systems/economics, Public Health, and Tobacco Products, are the core topics that constantly evolved between the years 2010 and 2018. Tobacco Use Disorder/therapy, Tobacco Use Disorder/epidemiology, Students/psychology, Students/statistics & numerical data, Adolescent Behavior/psychology, Nicotine/toxicity, Nicotinic Agonists/administration & dosage, and Electronic Nicotine Delivery Systems/legislation & jurisprudence can be considered as hot research topics in the analyzed period 2016–2018.

## References

[1] US Department of Health and Human Services (2016). E-cigarette use among youth and young adults: A report of the Surgeon General.

[2] Toy J, Dong F, Lee C (2017). Alarming increase in electronic nicotine delivery systems-related burn injuries: A serious unregulated public health issue. Am J Emerg Med.

[3] Gotts JE, Jordt SE, McConnell R, Tarran R (2019). What are the respiratory effects of e-cigarettes?. BMJ.

[4] National Academies of Sciences, Engineering, and Medicine (2018). Public health consequences of e-cigarettes.

[5] Walley SC, Wilson KM, Winickoff JP, Groner J (2019). A Public Health Crisis: Electronic Cigarettes, Vape, and JUUL. Pediatrics.

[6] Mays D, Smith C, Johnson AC, Tercyak KP, Niaura RS (2016). An experimental study of the effects of electronic cigarette warnings on young adult nonsmokers’ perceptions and behavioral intentions. Tob Induc Dis.

[7] Layden JE, Ghinai I, Pray I (2020). Pulmonary Illness Related to E-Cigarette Use in Illinois and Wisconsin - Final Report. N Engl J Med.

[8] Henry TS, Kanne JP, Kligerman SJ (2019). Imaging of Vaping-Associated Lung Disease. N Engl J Med.

[9] Ding N, Sang Y, Chen J (2019). Cigarette Smoking, Smoking Cessation, and Long-Term Risk of 3 Major Atherosclerotic Diseases. J Am Coll Cardiol.

[10] Hasan KM, Friedman TC, Shao X (2019). E-cigarettes and Western Diet: Important Metabolic Risk Factors for Hepatic Diseases. Hepatology.

[11] Johnson AL, McLeish AC, Shear PK, Sheth A, Privitera M (2019). The role of cigarette smoking in epilepsy severity and epilepsy-related quality of life. Epilepsy Behav.

[12] Cullen KA, Ambrose BK, Gentzke AS, Apelberg BJ, Jamal A, King BA (2018). Notes from the Field: Use of Electronic Cigarettes and Any Tobacco Product Among Middle and High School Students - United States, 2011-2018. MMWR Morb Mortal Wkly Rep.

[13] Gentzke AS, Creamer M, Cullen KA (2019). Vital Signs: Tobacco Product Use Among Middle and High School Students - United States, 2011-2018. MMWR Morb Mortal Wkly Rep.

[14] Veliz P, Eisman A, McCabe SE, Evans-Polce R, McCabe VV, Boyd CJ (2019). E-Cigarette Use, Polytobacco Use, and Longitudinal Changes in Tobacco and Substance Use Disorder Symptoms Among U.S. Adolescents. J Adolesc Health.

[15] Kim JS, Kim K (2019). Electronic cigarette use and suicidal behaviors among adolescents. J Public Health (Oxf).

[16] Mathers M, Toumbourou JW, Catalano RF, Williams J, Patton GC (2006). Consequences of youth tobacco use: a review of prospective behavioural studies. Addiction.

[17] Briganti M, Delnevo CD, Brown L, Hastings SE, Steinberg MB (2019). Bibliometric Analysis of Electronic Cigarette Publications: 2003-2018. Int J Environ Res Public Health.

[18] Zyoud SH, Al-Jabi SW, Sweileh WM (2014). Worldwide research productivity in the field of electronic cigarette: a bibliometric analysis. BMC Public Health.

[19] Donoghue JC (2012). Striving for zero tolerance of lateral violence. Ala Nurse.

[20] Zhang Q, Yue Y, Shi B, Yuan Z (2019). A Bibliometric Analysis of Cleft Lip and Palate-Related Publication Trends From 2000 to 2017. Cleft Palate Craniofac J.

[21] Viedma-Del-Jesus MI, Perakakis P, Munoz MA, Lopez-Herrera AG, Vila J (2011). Sketching the first 45 years of the journal Psychophysiology (1964-2008): a co-word-based analysis. Psychophysiology.

[22] Callon M (2015). [How to make useful the public debates]. Med Sci (Paris).

[23] Polosa R, Benowitz NL (2011). Treatment of nicotine addiction: present therapeutic options and pipeline developments. Trends Pharmacol Sci.

[24] Bhatt SP, Kim YI, Harrington KF (2018). Smoking duration alone provides stronger risk estimates of chronic obstructive pulmonary disease than pack-years. Thorax.

[25] Balata H, Fong KM, Hendriks LE (2019). Prevention and Early Detection for NSCLC: Advances in Thoracic Oncology 2018. J Thorac Oncol.

[26] Maina WK, Kitonyo R, Ogwell AE (2013). Using findings from a public opinion poll to build political support for tobacco control policy in Kenya. Tob Control.

[27] Etter JF, Bullen C, Flouris AD, Laugesen M, Eissenberg T (2011). Electronic nicotine delivery systems: a research agenda. Tob Control.

[28] Brown J, Beard E, Kotz D, Michie S, West R (2014). Real-world effectiveness of e-cigarettes when used to aid smoking cessation: a cross-sectional population study. Addiction.

[29] Nonnemaker J, Kim AE, Lee YO, MacMonegle A (2016). Quantifying how smokers value attributes of electronic cigarettes. Tob Control.

[30] Husari A, Shihadeh A, Talih S, Hashem Y, El Sabban M, Zaatari G (2015). Acute Exposure to Electronic and Combustible Cigarette Aerosols: Effects in an Animal Model and in Human Alveolar Cells. Nicotine Tob Res.

[31] Schraufnagel DE, Blasi F, Drummond MB (2014). Electronic cigarettes. A position statement of the forum of international respiratory societies. Am J Respir Crit Care Med.

[32] Fetterman JL, Weisbrod RM, Feng B (2018). Flavorings in Tobacco Products Induce Endothelial Cell Dysfunction. Arterioscler Thromb Vasc Biol.

[33] Staudt MR, Salit J, Kaner RJ, Hollmann C, Crystal RG (2018). Altered lung biology of healthy never smokers following acute inhalation of E-cigarettes. Respir Res.

[34] Schaal CM, Bora-Singhal N, Kumar DM, Chellappan SP (2018). Regulation of Sox2 and stemness by nicotine and electronic-cigarettes in non-small cell lung cancer. Mol Cancer.

[35] Ben Taleb Z, Ebrahimi Kalan M (2018). World Vapor Expo 2017: e-cigarette marketing tactics. Tob Control.

[36] Hudmon KS, Elkhadragy N, Kusynova Z, Besancon L, Brock TP, Corelli RL (2017). Global sale of tobacco products and electronic nicotine delivery systems in community pharmacies. Tob Control.

[37] Williams RS, Derrick J (2018). Content analysis of e-cigarette products, promotions, prices and claims on Internet tobacco vendor websites, 2013-2014. Tob Control.

[38] Garcia R, Sidhu A, Allem JP, Baezconde-Garbanati L, Unger JB, Sussman S (2016). Marketing activities of vape shops across racial/ethnic communities. Tob Prev Cessat.

[39] Sears C, Hart J, Walker K, Lee A, Keith R, Ridner S (2016). A Dollars and “Sense” Exploration of Vape Shop Spending and E-cigarette Use. Tob Prev Cessat.

[40] Reinhold B, Fischbein R, Bhamidipalli SS, Bryant J, Kenne DR (2017). Associations of attitudes towards electronic cigarettes with advertisement exposure and social determinants: a cross sectional study. Tob Induc Dis.

[41] Merrill JK, Alberg AJ, Goffin JR, Ramalingam SS, Simmons VN, Warren GW (2017). American Society of Clinical Oncology Policy Brief: FDA’s Regulation of Electronic Nicotine Delivery Systems and Tobacco Products. J Oncol Pract.

