# Supplementary material for: Electronic cigarettes: Emerging trends and research hotspots
Source: Tob Induc Dis. 2020 Mar 16;18:16. doi: 10.18332/tid/118719 (PMC7107908; doi:10.18332/tid/118719)
Supplement: Supplementary file 1 [file TID-18-16-s1.pdf]

Figure S1. Biclustering analysis of the top 15 high frequency major MeSH terms/MeSH subheadings and electronic cigarette articles from 2010 to 2012

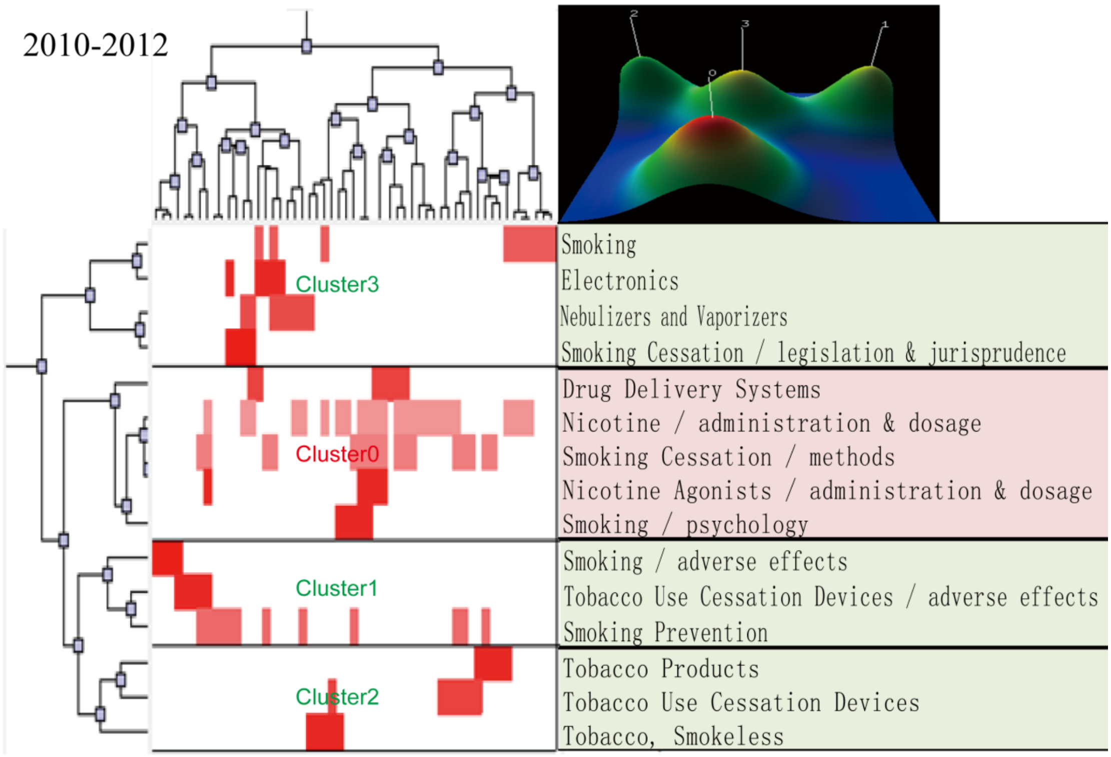

Figure S2. Biclustering analysis of the top 26 high frequency major MeSH terms/MeSH subheadings and electronic cigarette articles from 2013 to 2015.

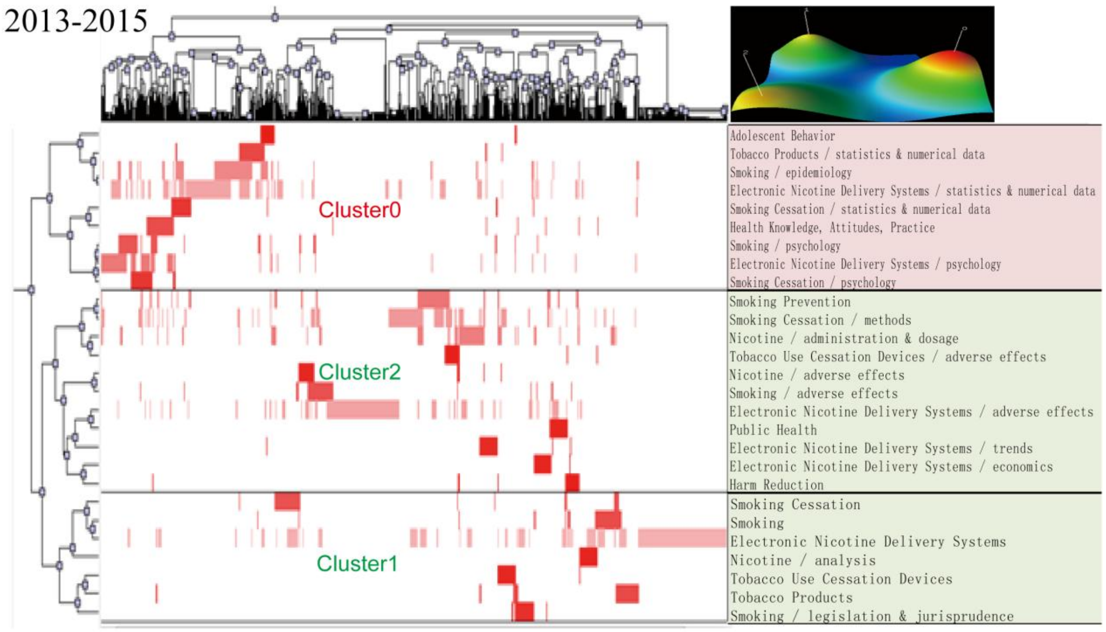

Figure S3. Biclustering analysis of the top 49 high frequency major MeSH terms/MeSH subheadings and electronic cigarette articles from 2016 to 2018

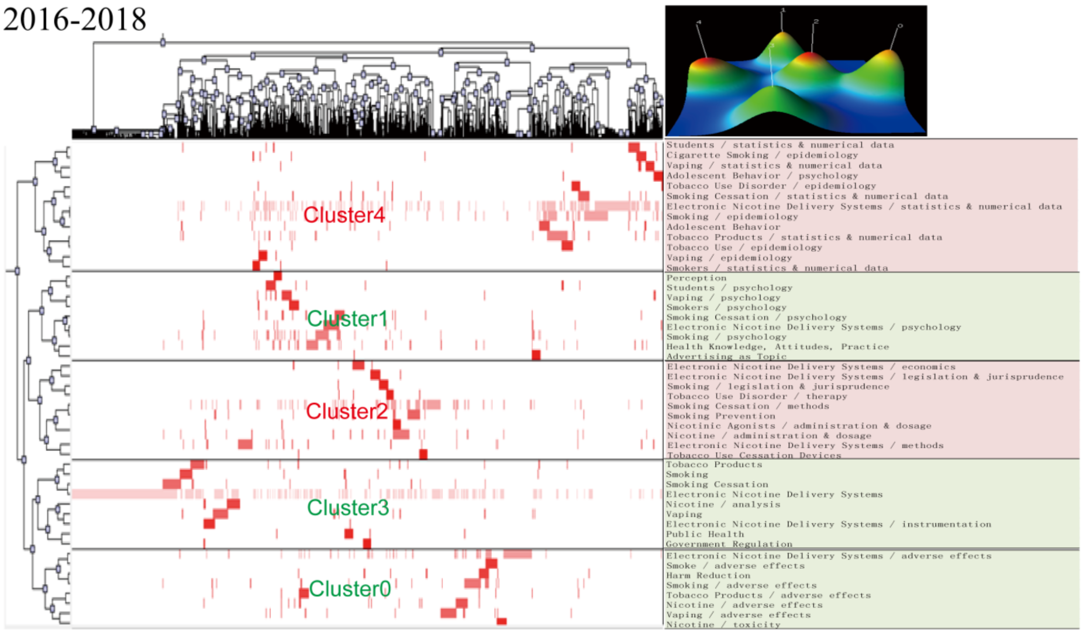

© 2020 Zhang Q. et al.
