# Supplementary material for: Electronic cigarettes: Emerging trends and research hotspots
Source: Tob Induc Dis. 2020 Mar 16;18:16. doi: 10.18332/tid/118719 (PMC7107908; doi:10.18332/tid/118719)
Supplement: Supplementary file 2 [file TID-18-16-s2.pdf]

Table S1 High-frequency MeSH terms/MeSH subheadings from the included papers on e-cigarette in 2010-2012, 2013-2015, 2016-2018

Period	Rank	MeSH terms/MeSH subheadings	Frequency of occurrence, n（%）
2010-2012			
	1	Nicotine/administration & dosage	25（10）
	2	Smoking Cessation/methods	17（6.8）
	3	Smoking Prevention	12（4.8）
	4	Smoking	10（4）
	5	Nebulizers and Vaporizers	8（3.2）
	6	Tobacco Use Cessation Devices	7（2.8）
	7	Drug Delivery Systems	7（2.8）
	8	Nicotinic Agonists/administration & dosage	5（2）
	9	Electronics	5（2）
	10	Tobacco Products	5（2）
	11	Tobacco Use Cessation Devices/adverse effects	5（2）
	12	Smoking/psychology	5（2）
	13	Tobacco, Smokeless	5（2）
	14	Smoking Cessation/legislation & jurisprudence	4（1.6）
	15	Smoking/adverse effects	4（1.6）
2013-2015			
	1	Electronic Nicotine Delivery Systems	281（7.2）
	2	Electronic Nicotine Delivery Systems/statistics & numerical data	209（5.3）
	3	Electronic Nicotine Delivery Systems/adverse effects	196（5.0）
	4	Smoking Cessation/methods	163（4.2）
	5	Smoking/epidemiology	118（3.0）
	6	Nicotine/administration & dosage	101（2.6）
	7	Electronic Nicotine Delivery Systems/psychology	91（2.3）
	8	Smoking Prevention	88（2.2）
	9	Smoking/psychology	55（1.4）
	10	Smoking Cessation	52（1.3）
	11	Smoking/adverse effects	51（1.3）
	12	Health Knowledge, Attitudes, Practice	49（1.2）
	13	Smoking	48（1.2）
	14	Tobacco Products	46（1.1）
	15	Tobacco Products/statistics & numerical data	45（1.1）
	16	Smoking Cessation/statistics & numerical data	36（0.9）
	17	Smoking Cessation/psychology	36（0.9）
	18	Smoking/legislation & jurisprudence	33（0.8）
	19	Electronic Nicotine Delivery Systems/trends	31（0.7）
	20	Tobacco Use Cessation Devices	29（0.7）
	21	Nicotine/adverse effects	29（0.7）

	22	Electronic Nicotine Delivery Systems/economics	28（0.7）
	23	Nicotine/analysis	28（0.7）
	24	Public Health	28（0.7）
	25	Harm Reduction	26（0.6）
	26	Tobacco Use Cessation Devices/adverse effects	25（0.6）
2016-2018			
	1	Electronic Nicotine Delivery Systems	658（9.9）
	2	Electronic Nicotine Delivery Systems/statistics & numerical data	356（5.3）
	3	Smoking/epidemiology	180（2.7）
	4	Smoking Cessation/methods	171（2.5）
	5	Electronic Nicotine Delivery Systems/adverse effects	148（2.2）
	6	Tobacco Products/statistics & numerical data	92（1.3）
	7	Smoking/psychology	90（1.3）
	8	Health Knowledge, Attitudes, Practice	90（1.3）
	9	Nicotine/administration & dosage	78（1.1）
	10	Smoking/adverse effects	76（1.1）
	11	Electronic Nicotine Delivery Systems/methods	66（0.9）
	12	Smoking Cessation	63（0.9）
	13	Smoking Prevention	59（0.8）
	14	Electronic Nicotine Delivery Systems/psychology	56（0.8）
	15	Vaping/adverse effects	56（0.8）
	16	Vaping	55（0.8）
	17	Tobacco Products	53（0.7）
	18	Smoking Cessation/statistics & numerical data	49（0.7）
	19	Nicotine/adverse effects	47（0.7）
	20	Smoking Cessation/psychology	45（0.6）
	21	Adolescent Behavior	44（0.6）
	22	Smoking	41（0.6）
	23	Students/statistics & numerical data	38（0.5）
	24	Nicotine/analysis	38（0.5）
	25	Vaping/psychology	38（0.5）
	26	Tobacco Products/adverse effects	36（0.5）
	27	Electronic Nicotine Delivery Systems/economics	36（0.5）
	28	Harm Reduction	35（0.5）
	29	Students/psychology	34（0.5）
	30	Smoke/adverse effects	32（0.4）
	31	Tobacco Use/epidemiology	32（0.4）
	32	Cigarette Smoking/epidemiology	32（0.4）
	33	Vaping/statistics & numerical data	30（0.4）
	34	Smokers/psychology	29（0.4）
	35	Electronic Nicotine Delivery Systems/instrumentation	29（0.4）
	36	Tobacco Use Disorder/epidemiology	28（0.4）

37	Electronic Nicotine Delivery Systems/legislation & jurisprudence	27（0.4）
38	Nicotine/toxicity	27（0.4）
39	Public Health	27（0.4）
40	Smoking/legislation & jurisprudence	25（0.3）
41	Adolescent Behavior/psychology	24（0.3）
42	Government Regulation	23（0.3）
43	Nicotinic Agonists/administration & dosage	23（0.3）
44	Advertising as Topic	23（0.3）
45	Tobacco Use Disorder/therapy	23（0.3）
46	Vaping/epidemiology	22（0.3）
47	Smokers/statistics & numerical data	21（0.3）
48	Perception	21（0.3）
49	Tobacco Use Cessation Devices	21（0.3）

MeSH = Medical Subject Headings

© 2020 Zhang Q. et al.
